# Embracing the systems complexity of microbial ecology and evolution: call for papers

**DOI:** 10.1128/msystems.00091-25

**Published:** 2025-02-20

**Authors:** Leonora Bittleston, Jack Gilbert, Jonathan Klassen, Rachel Mackelprang, Mark J. Mandel, Ryan J. Newton, Atmika Paudel, Alejandra Rodriguez-Verdugo, Ashley Shade, Roland C. Wilhelm, Ying Zhang

**Affiliations:** 1 Department of Biological Sciences, Boise State University, Boise, Idaho, USA; 2 Department of Pediatrics and Scripps Institution of Oceanography, University of California, San Diego, La Jolla, California, USA; 3 Department of Molecular and Cell Biology, University of Connecticut, Storrs, Connecticut, USA; 4 Department of Biological Sciences, California State University Northridge, Northridge, California, USA; 5 Department of Medical Microbiology & Immunology, University of Wisconsin Madison, Madison, Wisconsin, USA; 6 School of Freshwater Sciences, University of Wisconsin-Milwaukee, Milwaukee, Wisconsin, USA; 7 GenEndeavor LLC, Hayward, California, USA; 8 Department of Ecology and Evolutionary Biology, University of California Irvine, Irvine, California, USA; 9 Universite Claude Bernard Lyon 1, CNRS, INRAE, VetAgro Sup, Laboratoire d'Ecologie Microbienne LEM, CNRS UMR5557, INRAE UMR1418, Villeurbanne, France; 10 Department of Agronomy, Lilly Hall of Life Sciences, Purdue University, West Lafayette, Indiana, USA; 11 Department of Cell and Molecular Biology, College of the Environment and Life Sciences, University of Rhode Island, Kingston, Rhode Island, USA

## EDITORIAL

Microbial ecology and evolution are broad disciplines that seek to understand the underlying processes that drive the diversification, distributions, and dynamics of microbial cells, populations, communities, and ecosystems. Fundamentally, microbial ecology and evolution investigate complex systems. These systems include interactions among biotic and abiotic components within and across scales, over space and time, and from which biologically interesting properties emerge. Understanding the stochastic and deterministic processes governing microbial ecology and evolution can provide an essential framework for understanding microbial systems biology. With that framework, we are better positioned, for example, to decipher microbe-microbe and host-microbe interactions, selection and maintenance of diversity, resistance and resilience of microbiomes to perturbation, and ecosystem-level consequences of microbial responses to anthropogenic disturbances and climate change. Therefore, *mSystems* is issuing a call for papers to illuminate and promote the importance of microbial ecology and evolution as systems sciences.

*mSystems* welcomes original Research Articles, Methods and Protocols, Observations, Opinions/Hypotheses, Minireviews, and Perspective articles that elevate systems-level insights from microbial ecology and evolution. We especially encourage rigorous hypothesis-driven experiments that address ecological or evolutionary theories or consider factors of scale and outcomes of interacting components. Topics could examine feedback between microbes and their hosts/environments, biotic interactions, consequences for (eco)systems and functions, emergent properties, eco-evolutionary processes, underlying molecular mechanisms, and novel approaches to integrate, scale, or predict responses from quantitative data. We will consider studies that span the spectrum of complexity and controllability, from laboratory-scale experiments to *in situ* environmental or host-associated research, and we especially welcome studies that coalesce holistic and reductionist approaches. We encourage studies that feature the integration of different but complementary datatypes to enrich our understanding of systems-level microbial ecology and evolution. For evolution, this could include studies anchored to processes at the gene (e.g., microdiversity), genomic (e.g., pangenomics, ecotypic variation), and subsystems (e.g., metabolism, regulation) levels. For ecology, this could include processes scaled from interacting molecules (exometabolites, nucleic acids, enzymes) to ecosystem functions (biogeochemical cycles and process rates). In short, we want to receive your most ambitious and intricate research!

Ecological and evolutionary insights are often gained from meticulously executing methods that generate data aligned with the study’s scientific objective. Thus, we emphasize agnosticism in methodology as long the approaches and tools are robust and appropriate to the research questions. Similarly, we seek articles that generate systems-level insights. Observational studies that do not align with ecological or evolutionary questions may not match *mSystems* priorities (e.g., biodiversity inventories, new habitat descriptions, single-genome characterization).

*mSystems* eagerly welcomes your rigorous and innovative contributions that push the boundaries of systems research in microbial ecology and evolution.

